# Primary Prevention of Cardiocerebrovascular Diseases and Related Deaths According to Statin Type

**DOI:** 10.3390/ijerph17176309

**Published:** 2020-08-30

**Authors:** Joungyoun Kim, Hyeong-Seop Kim, Woojung Yang, Jae-woo Lee, Hee-Taik Kang

**Affiliations:** 1 Department of Information & Statistics, Chungbuk National University, Cheongju 28644, Korea; joungyoun@gmail.com (J.K.); 12__6452@naver.com (H.-S.K.); 2 Department of Family Medicine, Chungbuk National University Hospital, Cheongju 28644, Korea; kineto@naver.com (W.Y.); shrimp0611@gmail.com (J.-w.L.); 3 Department of Family Medicine, Chungbuk National University College of Medicine, Cheongju 28644, Korea

**Keywords:** HMG-CoA reductase inhibitors, pitavastatin, cardiovascular diseases, cerebrovascular diseases, hypercholesterolemia

## Abstract

(1) *Background*: Statin is the mainstay of treatment for the primary prevention of atherosclerotic cardiocerebrovascular diseases (CCVDs) in adults with hypercholesterolemia. This study aims to investigate the differences in effect on primary composite outcomes (CCVDs and CCVD-related deaths) among five statins in hypercholesterolemic individuals. (2) *Methods*: This retrospective study is based on the Korean National Health Insurance Service-National Health Screening Cohort. Participants, aged 40 to 69 years at baseline, were categorized into five statin-treated groups (pitavastatin, atorvastatin, rosuvastatin, simvastatin, and pravastatin) and two untreated groups (untreated hypercholesterolemia and no hypercholesterolemia). (3) *Results*: A total of 161,583 individuals was included. The median follow-up period was 8.2 years. Compared with the pitavastatin group, the hazard ratios (HRs; 95% confidence intervals (CIs)) for CCVDs and CCVD-related deaths of the atorvastatin, rosuvastatin, simvastatin, pravastatin, untreated hypercholesterolemia, and no-hypercholesterolemia groups were 0.969 (0.567–1.657), 0.988 (0.533–1.832), 0.862 (0.490–1.518), 0.906 (0.326–2.515), 2.665 (1.556–4.562), and 0.656 (0.388–1.110), respectively, in men and 1.124 (0.632–1.999), 1.119 (0.582–2.152), 1.324 (0.730–2.400), 1.023 (0.330–3.171), 2.650 (1.476–4.758), and 0.921 (0.522–1.625), respectively, in women, after being fully adjusted. (4) *Conclusions*: No significant differences among the five statins were observed, but there was an increased risk in untreated hypercholesterolemic individuals, for CCVDs and CCVDs-related deaths in individuals with hypercholesterolemia of either sex.

## 1. Introduction

Cardiovascular and cerebrovascular diseases are the second and fourth most common causes of death in Korea, respectively [1]. Therefore, the prevention of cardiocerebrovascular diseases (CCVDs) will help substantially reduce mortality and its associated public health burden. Most studies have indicated that statins reduce the risk of the first event in otherwise healthy individuals at high risk of atherosclerotic CCVDs [2,3,4]. Based on previous findings, most clinical practice guidelines recommend statin treatment for the primary prevention of atherosclerotic CCVDs in adults with hypercholesterolemia [5,6].

The main mechanism of statin to prevent CCVDs involves the reduction of blood cholesterol through inhibition of hepatic cholesterol synthesis by suppressing 3-hydroxy-3-methylglutaryl coenzyme A (HMG-CoA) reductase [7]. This inhibition leads to a compensatory increase of low-density lipoprotein (LDL)-cholesterol receptor synthesis through end-product feedback regulation by cholesterol [8]. As a consequence, increased LDL-cholesterol receptors more effectively lower serum LDL-cholesterol and total cholesterol levels. LDL-cholesterol-lowering efficacy varies among statins. Rosuvastatin is more potent than atorvastatin [9], and both these agents are significantly more potent than simvastatin, lovastatin, pravastatin, and fluvastatin [9,10]. Pitavastatin may be potentially as effective in lowering the LDL-cholesterol level as rosuvastatin [11].

However, there is a lack of evidence as to how effectively each statin can reduce overall atherosclerotic CCVDs and related deaths in individuals with hypercholesterolemia compared with other statins. The aim of this study is to investigate which among the five most commonly used statins is more effective in preventing atherosclerotic CCVDs and related deaths in individuals without a past history of CCVDs, after setting untreated hypercholesterolemia and no-hypercholesterolemia as control groups. To do so, we used two approaches. First, we looked at the differences among statin types by comparing incident primary composite outcomes of each (including CCVDs and CCVDs-related deaths) in hypercholesterolemic individuals based on the Korean National Health Insurance Service (NHIS)-National Health Screening Cohort (HEALS) data. Second, the efficacy of each statin was compared among the statin types after stratifying all the composite outcomes into cardiocerebrovascular, cardiovascular, and cerebrovascular diseases.

## 2. Materials and Methods

### 2.1. Study Population

The cohort consisted of 514,794 participants, a random sample from the 5.1 million health examinees between January 2002 and December 2003. The participants in the cohort were aged from 40 to 79 years at the end of December 2002. In addition, the cohort had available biennial national health screening results from 2002 to 2015. The database included diagnosis codes, medication prescriptions, death information, past histories of disease, and personal life habit information from the self-reported questionnaires.

**Figure 1:**
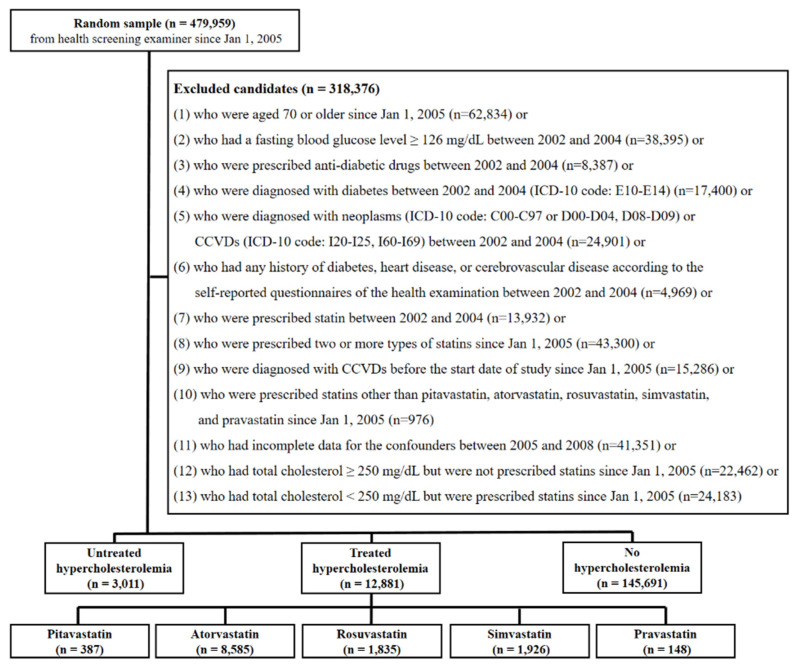
Flowchart of inclusion and exclusion criteria.

Figure 1 is a flowchart that shows how candidates were selected for the study. First, participants who underwent health screenings since 1 January 2005 (*n* = 479,959) were selected. Candidates were eliminated from the study if they satisfied any of the following conditions: (1) participants who were over 70 years old as 1 January 2005 (*n* = 62,834); (2) participants who had a fasting blood glucose level ≥126 mg/dL between 2002 and 2004 (*n* = 38,395); (3) participants who were prescribed antidiabetic drugs between 2002 and 2004 (*n* = 8387); (4) participants who were diagnosed with diabetes mellitus (DM; the 10th edition of the International Classification of Diseases (ICD-10) code: E11-E14) between 2002 and 2004 (*n* = 17,400); (5) participants who were diagnosed with any malignant neoplasms (ICD-10 code: C00-C97), in situ neoplasms (ICD-10 code: D00-D04, D08-D09), or CCVDs (ICD-10 code: I20-I25, I60-I69) between 2002 and 2004 (*n* = 24,901); (6) participants with any history of DM, heart disease, or cerebrovascular disease according to the self-reported questionnaires of the health examinations between 2002 and 2004 (*n* = 4969); (7) participants who were prescribed statins between 2002 and 2004 (*n* = 13,932); (8) participants who were prescribed two or more types of statins since 1 January 2005 (*n* = 43,300), (9) participants who were diagnosed with any CCVD before the start date of the study, 1 January 2005 (*n* = 15,286); (10) participants who were prescribed statins other than pitavastatin, atorvastatin, rosuvastatin, simvastatin, and pravastatin since 1 January 2005 (*n* = 976); (11) participants with any missing values in confounding variables between 2005 and 2008 (*n* = 41,351); (12) participants who had total cholesterol ≥250 mg/dL but were not prescribed statins since 1 January 2005 (*n* = 22,462); (13) participants who had total cholesterol <250 mg/dL but were prescribed statins since 1 January 2005 (*n* = 24,183). A final total of 161,583 participants was included in this study.

This research followed the 1964 Declaration of Helsinki, and the Institutional Review Board of Chungbuk National University approved this study (CBNU-201906-BMETC-870-01).

### 2.2. Definition of Cardiocerebrovascular Diseases

In this study, CCVD was defined based on ICD-10 codes (I20-I25, I60-I69). The primary outcomes of interest involved the main diagnosis of CCVDs (outpatient or hospitalization) or related deaths. For detailed analysis, we divided CCVDs into two groups: cardiovascular diseases (ICD-10 code: I20-I25) and cerebrovascular diseases (ICD-10 code: I60-I69). Cardiovascular diseases consisted of the following ICD-10 codes: I20, angina pectoris; I21, acute myocardial infarction; I22, subsequent myocardial infarction; I23, certain current complications following acute myocardial infarction; I24, other acute ischemic heart diseases; I25, chronic ischemic heart disease. Cerebrovascular diseases were comprised of the following ICD-10 codes: I60, subarachnoid hemorrhage; I61, intracerebral hemorrhage; I62, other nontraumatic intracranial hemorrhage; I63, cerebral infarction; I64, stroke, not specific as hemorrhage or infarction; I65, occlusion and stenosis of precerebral arteries, not resulting in cerebral infarction; I66, occlusion and stenosis of cerebral arteries, not resulting in cerebral infarction; I67, other cerebrovascular diseases; I68, cerebrovascular disorders in diseases classified elsewhere; I69, sequelae of cerebrovascular disease.

### 2.3. Hypercholesterolemia, Statin Types, and Study Periods

Our primary concern was hypercholesterolemic patients who were likely to take statin. In this study, a hypercholesterolemic patient was defined if he/she had total cholesterol levels ≥250 mg/dL and also had been prescribed statin since 1 January 2005. To identify the effect of statin on hypercholesterolmia, the hypercholesterolmic patients were divided into two groups, depending on whether the statin prescription days were 30 days or longer during the entire study period: (1) the treated hypercholesterolmic patients who were prescribed statin for 30 days or longer, and (2) the untreated hypercholesterolemic patients who had been prescribed statin for less than 30 days. The treated hypercholesterolemic patients were further divided into five groups according to the type of statin taken: (1) pitavastatin, (2) atorvastatin, (3) rosuvastatin, (4) simvastatin, and (5) pravastatin. Other types of statin were not available due to very small sample sizes or a limited number of events. In addition, individuals who took two or more types of statins were excluded. For healthy control, no hypercholesterolemia group were defined as individuals who had total cholesterol <250 mg/dL and also had never been prescribed any kinds of statin during the entire study period.

For hypercholesterolemic patients, the start date of the study was defined as the most recent of two dates: (1) the first date of health screening with total cholesterol level ≥250 mg/dL, or (2) initial prescription date for statin. For no hypercholesterolemic group, the start date was the first health screening date since 2005. If a patient experienced either CCVDs or related deaths, that day was the end date of the study. Otherwise, the latest of the following dates was the study end date: (1) the last date of health screening, (2) the last date of outpatient visit, or (3) the last date of statin intake.

### 2.4. Potential Confounders

In this study, the data on the confounding variables were extracted from the oldest health screening records between 2005 and 2008. The confounding variables were age, body mass index (BMI), systolic blood pressure (SBP), glucose, total cholesterol, alanine aminotransferase (ALT), DM history, smoking status, alcohol status, physical activity, and income status. Age, BMI, SBP, glucose, total cholesterol, and ALT levels were continuous variables, while the others were categorical variables. Most categorical variables were from self-reported questionnaires. Past history of DM was categorized into “yes” and “no”. Smoking status was divided into two groups: nonsmokers (who have never smoked) and smokers (who have smoked in the past or currently smoke). Alcohol status was stratified to rare (less than twice a month), sometimes (twice a month to twice a week), and often (more than twice a week). Physical activity was classified into three groups: rare (no exercise), sometimes (exercise between one and four times per week), and regular (exercise five times or more per week). Income status was stratified into three groups: low (0–<3rd decile), middle (3rd–<7th decile), and high (7th–10th decile).

### 2.5. Statistical Analysis

The statistical summary is presented as mean ± standard error (SE) for continuous variables and as number (percentage, %) for categorical variables. For each event, to compare the effects of each statin, ANOVA and Fisher’s exact test were performed. The Kaplan–Meier method and log-rank test were employed for nonparametric estimation and test of survival rates. Cox proportional hazard (PH) regression models were used to calculate hazard ratios (HRs) and 95% confidential intervals (CIs) for incident outcomes, after controlling for confounding factors. The reference for the above analyses was pitavastatin. Cox–PH models were classified into three levels depending on the number of confounding variables: (1) Model 1—only age, (2) Model 2—age, smoking status, alcohol status, and physical activity, (3) Model 3—past history of DM, income status, BMI, SBP, ALT, and total cholesterol, in addition to the variables in Model 2. All *p*-values are two-sided and <0.05 was regarded as statistically significant. The statistical package SAS enterprise guide version 7.1 (SAS Inc., Cary, NC, USA) and R studio version 3.3.3 were used to perform the analyses in this study.

## 3. Results

Of the final total of 161,583 participants (92,452 men and 69,131 women), 22,044 cases of overall CCVDs and CCVD-related deaths (13,524 men and 8520 women) occurred during the study period, accounting for 13.64% (14.63% in men and 12.32% in women) of all participants. The median follow-up duration was 8.2 years.

The baseline characteristics of the study participants, according to statin, are summarized in Table 1. Individuals treated with simvastatin were the oldest in both sexes among statin groups, while male pravastatin and female rosuvastatin groups were the youngest. BMI was highest in the pravastatin group in both sexes. SBP was highest in male pitavastatin and female pravastatin groups and lowest in male pravastatin and female rosuvastatin groups. Glucose level was highest in male pitavastatin and female simvastatin groups. Total cholesterol level was highest in the simvastatin group in both sexes and lowest in male pravastatin and female rosuvastatin groups among statin groups. The prevalence of DM was highest in male pravastatin and female simvastatin groups.

**Figure 2:**
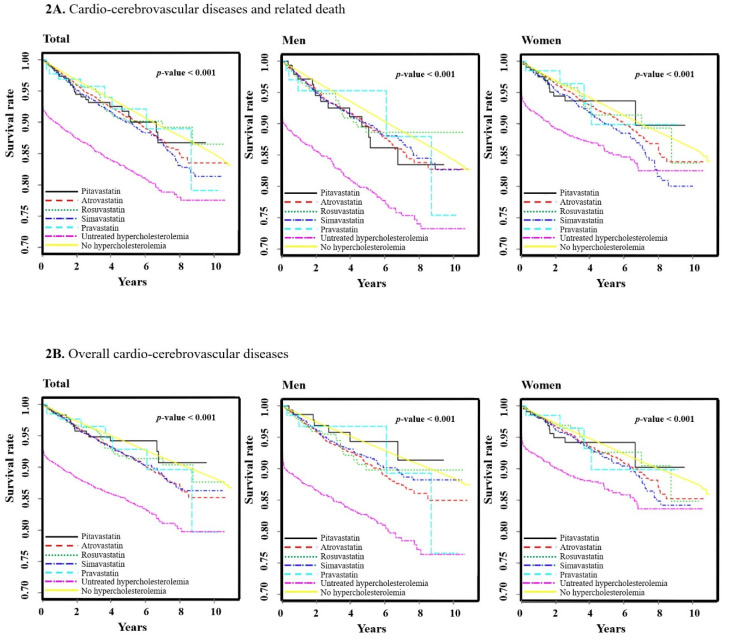

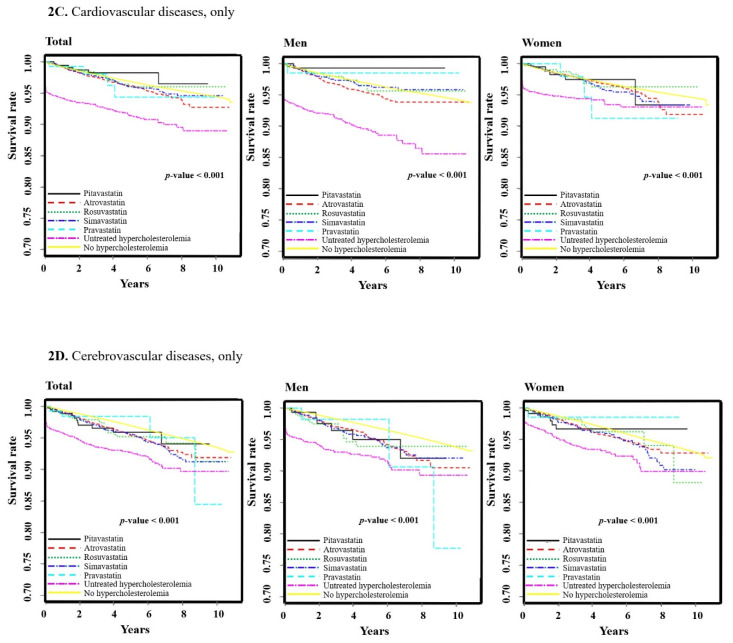
Kaplan–Meier estimates for the development of cardiocerebrovascular diseases and related deaths according to statin usage. (**A**) Cardiocerebrovascular diseases and related death. (**B**) Overall cardiocerebrovascular diseases. (**C**) Cardiovascular diseases only. (**D**) Cerebrovascular diseases only. *p*-Values are from log-rank tests.

The survival analysis was performed by the Kaplan–Meyer method and log-rank test to estimate the effects of the seven groups, including five statins, on the occurrence of CCVDs and CCVD-related deaths in Figure 2 (2A, overall CCVDs and CCVD-related deaths; 2B, overall CCVDs; 2C, cardiovascular diseases only; 2D, cerebrovascular diseases only). Significant differences in the incidence of the primary outcomes (CCVDs and CCVD-related deaths) or subgroups of CCVDs, according to statin type, were observed in either sex (all log-rank test *p*-Values <0.001).

The findings of the Cox-PH models for the primary composite outcomes (CCVDs and CCVDs-related deaths) are presented in Table 2. Compared with the pitavastatin group, the HRs (95% CIs) for the primary composite outcomes of the atorvastatin, rosuvastatin, simvastatin, pravastatin, untreated hypercholesterolemia, and no-hypercholesterolemia groups were 0.987 (0.578–1.688), 0.991 (0.535–1.838), 0.877 (0.499–1.544), 0.897 (0.323–2.489), 2.629 (1.536–4.500), and 0.697 (0.412–1.177), respectively, in men and 1.124 (0.632–1.998), 1.082 (0.563–2.080), 1.314 (0.724–2.383), 0.995 (0.321–3.085), 2.560 (1.426–4.596), and 0.991 (0.563–1.747), respectively, in women, after adjusting for age (Model 1). After fully adjusting for age, smoking status, drinking status, physical activity, BMI, SBP, total cholesterol, ALT, economic status, and DM history, the HRs (95% CIs) of atorvastatin, rosuvastatin, simvastatin, pravastatin, untreated hypercholesterolemia, and no-hypercholesterolemia groups were 0.969 (0.567–1.657), 0.988 (0.533–1.832), 0.862 (0.490–1.518), 0.906 (0.326–2.515), 2.665 (1.556–4.562), and 0.656 (0.388–1.110), respectively, in men and 1.124 (0.632–1.999), 1.119 (0.582–2.152), 1.324 (0.730–2.400), 1.023 (0.330–3.171), 2.650 (1.476–4.758), and 0.921 (0.522–1.625), respectively, in women (Model 3). Among the adjusted confounding factors, age, smoking status (ever vs. never), SBP, ALT, and DM were positively associated with primary composite outcomes (CCVDs and related deaths), while alcohol drinking, physical activity, and total cholesterol were inversely associated in both sexes (Table S1). In women, higher BMI and economic status increased the risks of primary composite outcomes, while higher total cholesterol levels reduced the risks (Table S1).

We conducted two additional analyses on (1) individuals with DM and (2) individuals regardless of DM history, leaving all the other inclusion and exclusion criteria the same. In each analysis, all results from the log-rank test were highly significant (*p*-values < 0.001; Figures S1 and S2). These imply that at least two groups among the seven groups have significantly different survival curve estimates. In the fully adjusted Cox–PH analysis, only the untreated hypercholesterolemia group showed a marginally significant risk of CCVDs and related deaths in Table S2 and a highly significant risk in Table S3.

The associations between the seven groups (five statin groups, untreated hypercholesterolemia, and no hypercholesterolemia) and the subgroup events of CCVDs are shown in Table 3. Cox–PH regression models were conducted after stratifying into overall CCVDs, cardiovascular diseases, and cerebrovascular diseases. Compared with the pitavastatin group, HRs (95% CIs) for overall CCVDs of the atorvastatin, rosuvastatin, simvastatin, pravastatin, untreated hypercholesterolemia, and no-hypercholesterolemia groups were 1.703 (0.804–3.609), 1.629 (0.717–3.700), 1.379 (0.632–3.007), 1.435 (0.420–4.903), 4.830 (2.277–10.244), and 1.004 (0.478–2.110), respectively, in males and 1.105 (0.606–2.015), 1.035 (0.520–2.060), 1.187 (0.635–2.220), 1.108 (0.353–3.480), 2.687 (1.459–4.950), and 0.864 (0.477–1.564), respectively, in females. HRs (95% CIs) for cardiovascular diseases and cerebrovascular diseases of the four types of statins were not statistically significant, while HRs of untreated hypercholesterolemia were significantly higher than those of the pitavastatin group, after being fully adjusted.

## 4. Discussion

In this study, we found no significant difference in the prevention of CCVDs and CCVD-related deaths among the seven groups, including five statins, in either sex. Even after stratifying all CCVDs into three subgroups (overall cardiocerebrovascular, cardiovascular, and cerebrovascular diseases), the five statins exhibited similar effects on the risk for subgroups of atherosclerotic CCVDs. However, untreated hypercholesterolemia increased the risk of CCVDs and related deaths in both sexes.

Statins were classified into three groups according to LDL-cholesterol-lowering efficacy. The 2013 American College of Cardiology/American Heart Association (ACC/AHA) guidelines recommend that the appropriate intensity of statins be chosen for individuals who have a higher risk for atherosclerotic CCVDs [6]. However, evidence to directly compare how efficiently individual statin types prevent the primary composite outcomes (CCVDs and related deaths) is rare [12]. In addition, although pitavastatin is potent for lowering blood total cholesterol, LDL-cholesterol, and triglyceride [13], it is classified as a moderate- to low-intensity statin [6]. Thus, we sought to investigate whether pitavastatin differed from other commonly prescribed statins in preventing the primary composite outcomes in hypercholesterolemic patients without apparent CCVDs.

High-density lipoprotein (HDL)-cholesterol mediates reverse transport of cholesterol from cells of the arterial wall to the liver and steroidogenic organs [14]. Most statins modestly raise the HDL-cholesterol level even though they mainly reduce the risk of CCVDs through HMG-CoA reductase inhibition and LDL-cholesterol-lowering mechanisms [15]. However, the efficacy in reducing LDL-cholesterol and increasing HDL-cholesterol varies from statin to statin. Pitavastatin is classified as a moderate- to low-intensity statin based on LDL-cholesterol-lowering ability, according to the 2013 ACC/AHA guidelines, but its effect on HDL-cholesterol elevation is better than those of atorvastatin, rosuvastatin, and simvastatin [15,16,17]. In addition, pitavastatin has a greater PH index (Δ plaque volume/Δ HDL-cholesterol), which represents the change in plaque volume induced by a 1% increase in HDL-cholesterol compared to other types of statin. This indicates that pitavastatin can more efficiently reduce atherosclerotic plaques [18]. According to these theoretical backgrounds, we hypothesized that pitavastatin could result in better primary composite outcomes or secondary outcomes. However, there were no significant differences among the five types of statins in preventing primary composite outcomes in this study.

The reason for the lack of differences in preventive effects may be due to the more complicated mechanisms of statins that cause a reduction of CCVDs and related deaths in apparently healthy individuals beyond LDL-cholesterol reduction and HDL-cholesterol elevation [19,20]. First, statins are known to be pluripotent in modulating cell signaling and reducing oxidative stress and inflammation. Statins inhibit the production of isoprenoid intermediates in the cholesterol biosynthetic pathway. Post-translational prenylation of small GTP-binding proteins such as Rho and its downstream effector NADPH oxidase are modified by statins [7,21]. Additionally, statins reduce proinflammatory processes such as the expression of adhesion molecules, particularly in monocytes [22]. Second, the participants’ condition should be considered. Since the data used in this study were from a real-world setting, the types of statins were decided based on individual risks of atherosclerotic CCVDs according to the 2013 ACC/AHA guidelines. Participants at higher risk of CCVDs may be prescribed a high-intensity statin. In addition, apparently healthy participants, even with hypercholesterolemia, may be at very low risk of developing CCVDs and related deaths. Third, information on participants’ lifestyle behaviors, such as dietary patterns, was not fully controlled because they were not available in the NHIS-HEALS database. These factors can affect these null associations between statin types and primary composite outcomes.

There are several strengths that distinguish this study from previous studies. First, we used data from a large population provided by NHIS-HEALS, based on real-world measurements in a clinical setting. Korean public authorities recommend obligatory medical insurance to cover the entire Korean population, including individuals of low socioeconomic status. Therefore, the NHIS-HEALS data well represent the entire Korean population. Second, national health insurance claim data include diagnosis and prescriptions. In addition, the Korean Ministry of Health and Welfare (MOHW) strongly recommends that all adults aged 40 years or older undergo public health examination services, including health-related questionnaires and several blood tests such as lipid profiling. Almost all claim data covered by the Korean NHIS are under the control of the Korean MOHW in order to assess and reimburse insurance claims from medical institutions. Therefore, recall bias is minimized. Third, socioeconomic status is a major indicator of healthcare accessibility and strongly affects patients’ clinical outcomes. We adjusted for monthly household income to control for health inequity based on socioeconomic status. Fourth, further analyses were conducted after the entire population was stratified into overall cardiocerebrovascular, cardiovascular, and cerebrovascular events to examine the association of statin types and subgroup events of CCVDs.

This study has some limitations that should be considered when interpreting this study. First, although several potential confounding factors were adjusted for, we could not completely control for several residual potential confounders such as genetic or familial vulnerabilities. We also could not include any lipid-lowering agents other than statins as confounders because of limited data. Second, there is the possibility of selection bias. Of the initial 479,959 people, only 12,881 single-statin users were included. It is possible that the exclusion of many participants led to a selection bias in this study. In addition, there were large differences in sample size. Propensity score matching is often used to avoid potential bias due to unbalanced sample size. However, simultaneous propensity score matching would result in significant sample size losses in the pitavastatin and pravastatin groups. These losses could lower the statistical power due to insufficient sample size and lead to a null association. To control for differences in several variables, specifically age and sex, age was adjusted for in Cox-PH regression models, while the entire population was stratified by sex in the final model. Third, the study population almost entirely belonged to the Korean ethnic group. Therefore, these findings may not apply to other racial and ethnic groups. Fourth, large-scale clinical trials are needed to compare the primary preventive effects of each statin type on CCVDs, as the number of participants in each statin group in this study is relatively small. Fifth, people at high risk of CCVDs might be excluded because individuals who were prescribed two or more types of statins during the entire study were ruled out. When at high risk, people are more likely to switch from lower-intensity statins to higher-intensity statins than to remain with a lower-intensity statin over time. Thus, to estimate future CCVD risk using Framingham risk scores or atherosclerotic cardiovascular disease risk estimators, it is very important to minimize potential biases. Unfortunately, HDL-cholesterol and LDL-cholesterol data were not available for the NHIS-HEALS cohort. We could not calculate future CCVD risk. Instead, strict exclusion criteria and a sufficient wash-out period were adopted to minimize possible biases. If a change in LDL-cholesterol and HDL-cholesterol was measured, we would have a better understanding of the efficacy of individual statins in preventing the primary composite outcomes.

## 5. Conclusions

In conclusion, no significant differences of incidence of CCVDs and related deaths according to statin type were observed in either sex in this study. However, untreated hypercholesterolemia increased risks for CCVDs and related deaths. Further clinical trials to compare the beneficial effects of each statin type on CCVDs and related deaths are required.

## Figures and Tables

**Table 1:** Baseline characteristics according to statin type.

Male	Pitavastatin	Atorvastatin	Rosuvastatin	Simvastatin	Pravastatin	Untreated Hypercholesterolemia	No Hypercholesterolemia	*p*-Value
Number (*n*)	162	3457	776	830	72	1512	85,643	
Age, years	50.9 ± 5.7	51.1 ± 5.9	50.3 ± 5.5	51.7 ± 6.4	49.7 ± 5.7	50.5 ± 5.9	52.2 ± 7.0	<0.001
BMI, kg/m^2^	24.4 ± 2.8	24.5 ± 2.7	24.5 ± 2.6	24.3 ± 2.7	24.8 ±2.6	24.2 ± 2.6	23.7 ±2.7	<0.001
SBP, mmHg	128.4 ± 17.0	128.0 ± 15.9	126.8 ± 14.6	128.0 ± 16.2	125.0 ± 16.4	124.7 ± 14.4	124.8 ± 15.3	<0.001
Glucose, mg/dL	97.7 ± 16.7	96.3 ±18.2	95.8 ± 16.0	96.6 ± 17.4	97.1 ± 13.7	95.5 ± 16.5	93.9 ± 16.6	<0.001
Total cholesterol, mg/dL	234.5 ± 36.4	235.3 ± 33.1	232.1 ± 33.0	239.6 ± 35.5	230.7 ± 32.7	234.0 ± 36.6	183.4 ± 27.3	<0.001
ALT, IU/L	31.8 ± 24.9	30.4 ± 19.0	30.4 ± 18.7	30.8 ± 16.9	29.2 ± 15.7	30.2 ± 20.0	27.5 ± 21.2	<0.001
DM, *n* (%)	7 (4.3)	237 (6.9)	40 (5.2)	53 (6.4)	6 (8.3)	47 (3.1)	4884 (8.3)	<0.001
Ever smokers, *n* (%)	78 (48.1)	1915 (55.4)	419 (54.0)	484 (58.3)	34 (47.2)	848 (56.1)	43,027 (50.2)	<0.001
Drinking status, *n* (%)								<0.001
Rare	53 (32.7)	1157 (33.5)	277 (35.7)	266 (32.0)	27 (37.5)	514 (34.0)	31,758 (37.1)	
Sometimes	84 (51.9)	1742 (50.4)	382 (49.2)	407 (49.0)	32 (44.4)	724 (47.9)	39,773 (46.4)	
Often	25 (15.4)	558 (16.1)	117 (15.1)	157 (18.9)	13 (18.1)	274 (18.1)	14,112 (16.5)	
Physical activity, *n* (%)								0.321
Rare	59 (36.4)	1433 (41.5)	317 (40.9)	369 (44.5)	29 (40.3)	664 (43.9)	36,820 (43.0)	
Sometimes	89 (54.9)	1710 (49.5)	387 (49.9)	391 (47.1)	36 (50.0)	737 (48.7)	41,152 (48.1)	
Regular	14 (8.6)	314 (9.1)	72 (9.3)	70 (8.4)	7 (9.7)	111 (7.3)	7671 (9.0)	
Economic status, *n* (%)								0.357
Low	20 (12.3)	521 (15.1)	110 (14.2)	134 (16.1)	9 (12.5)	246 (16.3)	13,114 (15.3)	
Middle	54 (33.3)	1098 (31.8)	221 (28.5)	266 (32.0)	17 (23.6)	489 (32.3)	27,166 (31.7)	
High	88 (54.3)	1838 (53.2)	445 (57.3)	430 (51.8)	46 (63.9)	777 (51.4)	45,363 (53.0)	
**Female**	**Pitavastatin**	**Atorvastatin**	**Rosuvastatin**	**Simvastatin**	**Pravastatin**	**Untreated hypercholesterolemia**	**No hypercholesterolemia**	***p*-Value**
Number (*n*)	225	5128	1059	1096	76	1499	60,048	
Age, years	52.3 ± 6.2	52.4 ±6.0	51.5 ± 5.8	53.2 ± 6.3	51.9 ± 6.0	52.0 ± 6.1	52.3 ± 6.9	<0.001
BMI, kg/m^2^	23.7 ± 2.7	24.0 ± 2.9	23.6 ± 2.8	24.0 ± 2.7	24.2 ± 2.9	23.7 ± 2.9	23.3 ±2.8	<0.001
SBP, mmHg	122.1 ± 16.4	123.5 ± 16.3	121.8 ± 16.2	123.3 ± 16.6	124.0 ± 16.9	120.1 ± 15.8	119.8 ± 15.8	<0.001
Glucose, mg/dL	92.1 ± 13.8	92.2 ± 15.6	92.6 ± 14.6	93.2 ± 16.1	93.1 ± 14.7	91.9 ± 15.5	90.2 ± 13.6	<0.001
Total cholesterol, mg/dL	226.2 ± 33.8	228.7 ± 33.7	225.9 ± 34.1	234.7 ± 38.8	229.6 ± 31.9	225.6 ± 34.7	183.3 ± 27.0	<0.001
ALT, IU/L	20.5 ± 11.3	21.5 ± 13.5	20.4 ± 17.7	22.1 ± 12.4	21.5 ± 13.8	21.5 ± 15.8	20.2 ± 16.8	<0.001
DM, *n* (%)	14 (6.2)	430 (8.4)	64 (6.0)	100 (9.1)	4 (5.3)	69 (4.6)	3771 (6.3)	<0.001
Ever smokers, *n* (%)	7 (3.1)	98 (1.9)	17 (1.6)	30 (2.7)	0 (0.0)	40 (2.7)	1176 (2.0)	0.097
Drinking status, *n* (%)								0.013
Rare	176 (78.2)	4135 (80.6)	852 (80.5)	872 (79.6)	63 (82.9)	1171 (78.1)	48,806 (81.3)	
Sometimes	42 (18.7)	927 (18.1)	192 (18.1)	210 (19.2)	10 (13.2)	297 (19.8)	10,300 (17.2)	
Often	7 (3.1)	66 (1.3)	15 (1.4)	14 (1.3)	3 (3.9)	31 (2.1)	942 (1.6)	
Physical activity, *n* (%)								0.328
Rare	120 (53.3)	2858 (55.7)	570 (53.8)	616 (56.2)	47 (61.8)	836 (55.8)	34,056 (56.7)	
Sometimes	83 (36.9)	1795 (35.0)	386 (36.4)	359 (32.8)	23 (30.3)	525 (35.0)	20,196 (33.6)	
Regular	22 (9.8)	475 (9.3)	103 (9.7)	121 (11.0)	6 (7.9)	138 (9.2)	5796 (9.7)	
Economic status, *n* (%)								<0.001
Low	54 (24.0)	1454 (28.4)	294 (27.8)	302 (27.6)	20 (26.3)	432 (28.8)	15,444 (25.7)	
Middle	78 (34.7)	1780 (34.7)	379 (35.8)	400 (36.5)	25 (32.9)	496 (33.1)	20,449 (34.1)	
High	93 (41.3)	1894 (36.9)	386 (36.4)	394 (35.9)	31 (40.8)	571 (38.1)	24,155 (40.2)	

**Table 2:** Cox-proportional hazard regression model for composite outcomes (cardiocerebrovascular diseases and related deaths), compared with pitavastatin.

Model	HRs (95% CIs)	Men	Women
Model 1	Atorvastatin	0.987 (0.578–1.688)	1.124 (0.632–1.998)
	Rosuvastatin	0.991 (0.535–1.838)	1.082 (0.563–2.080)
	Simvastatin	0.877 (0.499–1.544)	1.314 (0.724–2.383)
	Pravastatin	0.897 (0.323–2.489)	0.995 (0.321–3.085)
	Untreated hypercholesterolemia	2.629 (1.536–4.500)	2.560 (1.426–4.596)
	No hypercholesterolemia	0.697 (0.412–1.177)	0.991 (0.563–1.747)
Model 2	Atorvastatin	0.965 (0.564–1.649)	1.125 (0.633–2.000)
	Rosuvastatin	0.973 (0.525–1.805)	1.086 (0.565–2.087)
	Simvastatin	0.846 (0.481–1.489)	1.310 (0.722–2.376)
	Pravastatin	0.876 (0.316–2.433)	1.004 (0.324–3.114)
	Untreated hypercholesterolemia	2.539 (1.483–4.347)	2.556 (1.424–4.589)
	No hypercholesterolemia	0.681 (0.403–1.151)	0.990 (0.562–1.745)
Model 3	Atorvastatin	0.969 (0.567–1.657)	1.124 (0.632–1.999)
	Rosuvastatin	0.988 (0.533–1.832)	1.119 (0.582–2.152)
	Simvastatin	0.862 (0.490–1.518)	1.324 (0.730–2.400)
	Pravastatin	0.906 (0.326–2.515)	1.023 (0.330–3.171)
	Untreated hypercholesterolemia	2.665 (1.556–4.562)	2.650 (1.476–4.758)
	No hypercholesterolemia	0.656 (0.388–1.110)	0.921 (0.522–1.625)

Model 1: adjusted for age. Model 2: adjusted for smoking status (ever and never smokers), drinking status (rare, sometimes, and often) and physical activity (rare, sometimes, and regular) in addition to the variable of Model 1. Model 3: adjusted for body mass index, systolic blood pressure, total cholesterol, ALT, economic status (low, middle, and high), and DM (yes or no), in addition to the variables of Model 2.

**Table 3:** Cox-proportional hazard regression model for other outcomes (cardiocerebrovascular diseases, cardiovascular diseases, or cerebrovascular diseases) compared with pitavastatin.

Outcome	HRs (95% CIs)	Men	Women
Cardiocerebrovascular diseases	Atorvastatin	1.703 (0.804–3.609)	1.105 (0.606–2.015)
	Rosuvastatin	1.629 (0.717–3.700)	1.035 (0.520–2.060)
	Simvastatin	1.379 (0.632–3.007)	1.187 (0.635–2.220)
	Pravastatin	1.435 (0.420–4.903)	1.108 (0.353–3.480)
	Untreated hypercholesterolemia	4.830 (2.277–10.244)	2.687 (1.459–4.950)
	No hypercholesterolemia	1.004 (0.478–2.110)	0.864 (0.477–1.564)
Cardiovascular diseases only	Atorvastatin	5.707 (0.798–40.832)	1.060 (0.434–2.588)
	Rosuvastatin	4.084 (0.531–31.414)	0.895 (0.319–2.510)
	Simvastatin	3.795 (0.514–28.012)	1.188 (0.469–3.009)
	Pravastatin	2.409 (0.151–38.510)	1.796 (0.429–7.516)
	Untreated hypercholesterolemia	18.612 (2.603–133.098)	2.867 (1.161–7.076)
	No hypercholesterolemia	3.736 (0.526–26.555)	0.917 (0.380–2.210)
Cerebrovascular diseases only	Atorvastatin	1.045 (0.461–2.369)	1.145 (0.508–2.584)
	Rosuvastatin	1.272 (0.508–3.186)	1.173 (0.465–2.955)
	Simvastatin	0.972 (0.412–2.292)	1.189 (0.510–2.770)
	Pravastatin	1.297 (0.324–5.187)	0.517 (0.062–4.297)
	Untreated hypercholesterolemia	2.508 (1.100–5.718)	2.527 (1.103–5.793)
	No hypercholesterolemia	0.555 (0.248–1.238)	0.824 (0.369–1.840)

Adjusted for age, smoking status (ever and never smokers), drinking status (rare, sometimes, and often), physical activity (rare, sometimes, and regular), body mass index, systolic blood pressure, total cholesterol, ALT, economic status (low, middle, and high), and DM (yes or no).

## Supplementary Materials

The following are available online at https://www.mdpi.com/1660-4601/17/17/6309/s1. Figure S1: Kaplan-Meier estimates for the development of cardiocerebrovascular diseases and related deaths according to statin usage among patients with diabetes. Figure S2: Kaplan-Meier estimates for the development of cardiocerebrovascular diseases and related deaths according to statin usage among individuals, regardless of diabetes. Table S1: Hazard ratios for composite outcomes (cardiocerebrovascular diseases and related deaths) of adjusted risk factors. Table S2: Cox-proportional hazard regression model for composite outcomes (cardiocerebrovascular diseases and related deaths) among patients with diabetes, compared with pitavastatin. Table S3: Cox-proportional hazard regression model for composite outcomes (cardiocerebrovascular diseases and related deaths) among individuals regardless of diabetes, compared with pitavastatin.

## Abbreviations

BMI	body mass index
SBP	systolic blood pressure
ALT	alanine transaminase
DM	diabetes mellitus
