# Supplementary material for: Primary Prevention of Cardiocerebrovascular Diseases and Related Deaths According to Statin Type
Source: Int J Environ Res Public Health. 2020 Aug 30;17(17):6309. doi: 10.3390/ijerph17176309 (PMC7503978; doi:10.3390/ijerph17176309)
Supplement: Supplementary file 1 [file ijerph-17-06309-s001.pdf]

Table S1. Hazard ratios for composite outcomes (cardio-cerebrovascular diseases and related deaths) of adjusted risk factors.

HRs (95% CIs)	Men	Women
Age (every 1 year)	1.070 (1.067–1.072)	1.062 (1.058–1.065)
Smoking status (ever vs never)	1.159 (1.118–1.201)	1.533 (1.340–1.753)
Drinking status		
Sometimes vs rare	0.947 (0.910–0.986)	0.949 (0.892–1.010)
Often vs rare	1.046 (0.996–1.098)	0.865 (0.718–1.040)
Physical activity		
Sometimes vs rare	0.900 (0.867–0.933)	0.951 (0.906–0.998)
Regular vs rare	0.914 (0.862–0.969)	0.967 (0.900–1.039)
Body mass index (every 1 kg/m^2)	0.997 (0.990–1.003)	1.018 (1.010–1.026)
Systolic blood pressure (every 1 mmHg)	1.054 (1.042–1.065)	1.026 (1.012–1.040)
Total cholesterol (every 1 mg/dL)	0.986 (0.980–0.992)	0.979 (0.971–0.987)
ALT (every 1 IU/L)	1.003 (1.002–1.003)	1.001 (1.000–1.002)
Economic status		
Middle vs low	0.994 (0.947–1.043)	1.077 (1.018–1.139)
High vs low	0.997 (0.951–1.046)	1.113 (1.054–1.176)
DM (yes vs no)	1.323 (1.247–1.404)	1.422 (1.326–1.525)

Adjusted for age, smoking status (ever and never smokers), drinking status (rare, sometimes, and often) and physical activity (rare, sometimes, and regular), body mass index, systolic blood pressure, total cholesterol, ALT, economic status (low, middle, and high), and DM (yes or no), in addition to seven groups (five statin types, untreated hypercholesterolemia, and no hypercholesterolemia groups).

Figure S1. Kaplan-Meier estimates for development of cardio-cerebrovascular diseases and related deaths according to statin usage among patients with diabetes.

1A. Cardio-cerebrovascular diseases and related death

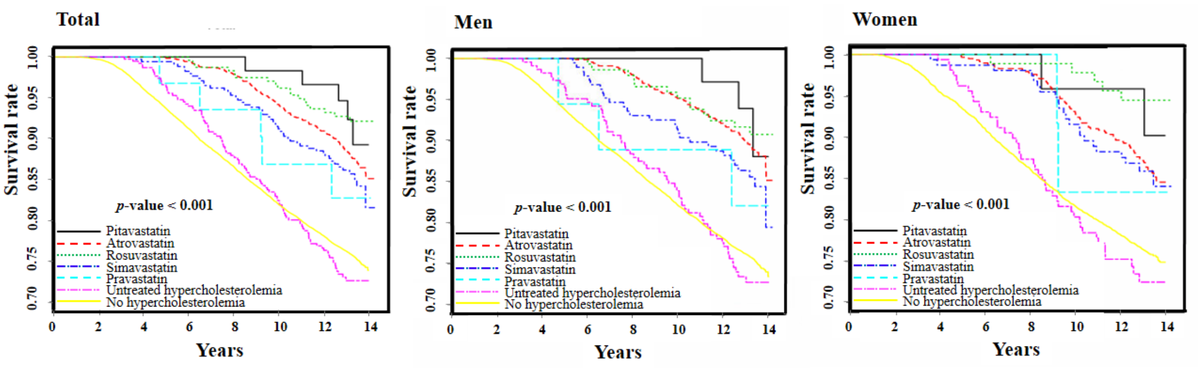

Figure S1. Kaplan-Meier estimates for development of cardio-cerebrovascular diseases and related deaths according to statin usage among patients with diabetes.

1B. Overall cardio-cerebrovascular diseases

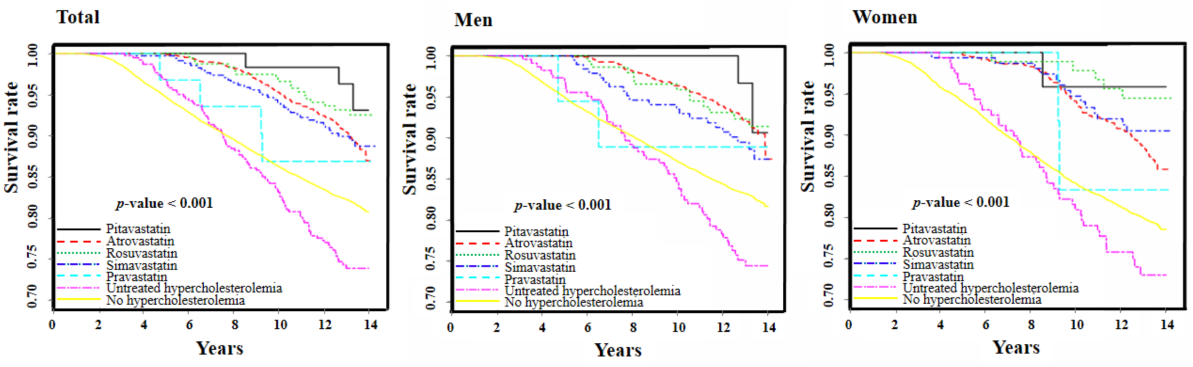

Figure S1. Kaplan-Meier estimates for development of cardio-cerebrovascular diseases and related deaths according to statin usage among patients with diabetes.

1C. Cardiovascular diseases, only

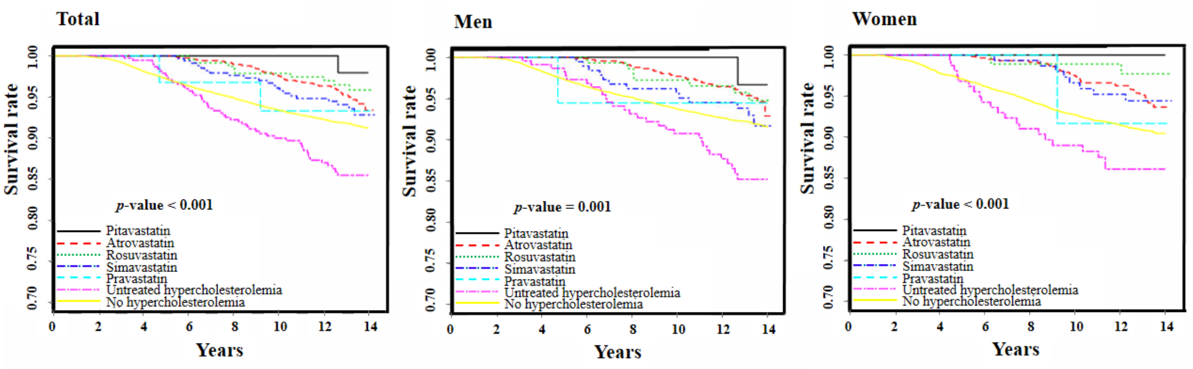

Figure S1. Kaplan-Meier estimates for development of cardio-cerebrovascular diseases and related deaths according to statin usage among patients with diabetes.

1D. Cerebrovascular diseases, only

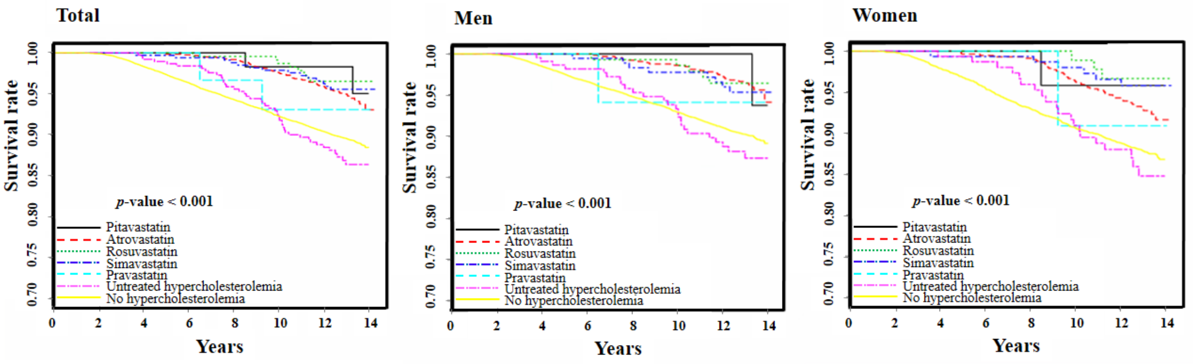

Figure 1. Kaplan-Meier estimates for development of cardio-cerebrovascular diseases and related deaths according to statin usage among patients with diabetes.

Table S2. Cox-proportional hazard regression model for composite outcomes (cardio-cerebrovascular diseases and related deaths) among patients with diabetes, compared with pitavastatin.

Model	HRs (95% CIs)	Men	Women
Model 1	Atorvastatin	1.164 (0.367–3.693)	1.433 (0.352–5.830)
	Rosuvastatin	0.993 (0.283–3.485)	0.595 (0.115–3.069)
	Simvastatin	1.624 (0.494–5.343)	1.477 (0.347–6.285)
	Pravastatin	1.912 (0.386–9.472)	2.046 (0.288–14.526)
	Untreated hypercholesterolemia	3.138 (0.984–10.011)	3.143 (0.761–12.987)
	No hypercholesterolemia	2.398 (0.773–7.440)	2.548 (0.636–10.202)
Model 2	Atorvastatin	1.160 (0.366–3.678)	1.421 (0.349–5.786)
	Rosuvastatin	0.994 (0.283–3.489)	0.592 (0.115–3.051)
	Simvastatin	1.632 (0.496–5.370)	1.470 (0.345–6.255)
	Pravastatin	1.788 (0.361–8.863)	1.942 (0.273–13.797)
	Untreated hypercholesterolemia	3.041 (0.953–9.700)	3.103 (0.751–12.825)
	No hypercholesterolemia	2.396 (0.772–7.436)	2.521 (0.630–10.100)
Model 3	Atorvastatin	1.158 (0.365–3.676)	1.409 (0.346–5.738)
	Rosuvastatin	1.009 (0.287–3.541)	0.596 (0.116–3.072)
	Simvastatin	1.654 (0.503–5.443)	1.469 (0.345–6.249)
	Pravastatin	1.874 (0.378–9.292)	1.968 (0.277–13.986)
	Untreated hypercholesterolemia	3.109 (0.974–9.923)	3.101 (0.750–12.816)
	No hypercholesterolemia	2.152 (0.693–6.687)	2.348 (0.584–9.435)

Model 1: adjusted for age; Model 2: adjusted for smoking status (ever and never smokers), drinking status (rare, sometimes, and often) and physical activity (rare, sometimes, and regular) in addition to variable of Model 1; Model 3: adjusted for body mass index, systolic blood pressure, total cholesterol, ALT, and economic status (low, middle, and high), in addition to variables of Model 2.

Figure S2. Kaplan-Meier estimates for development of cardio-cerebrovascular diseases and related deaths according to statin usage among individuals regardless of diabetes.

2A. Cardio-cerebrovascular diseases and related death

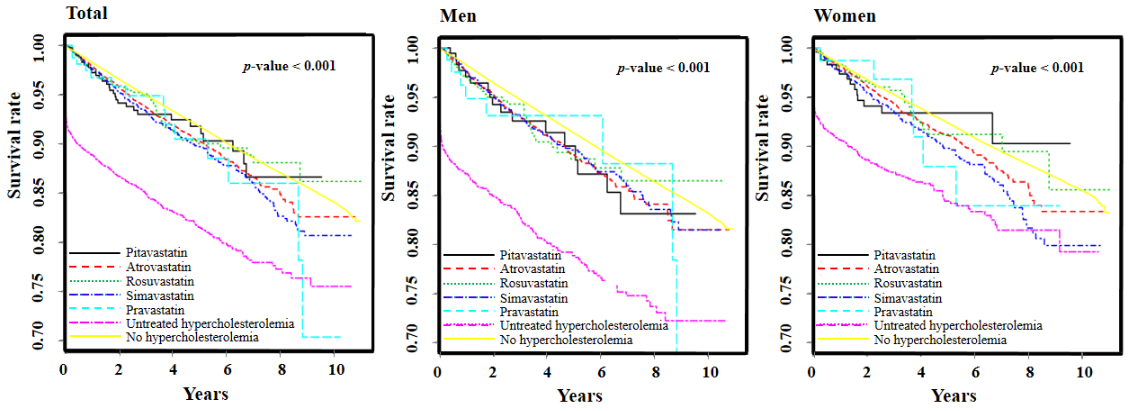

Figure S2. Kaplan-Meier estimates for development of cardio-cerebrovascular diseases and related deaths according to statin usage among individuals regardless of diabetes.

2B. Overall cardio-cerebrovascular diseases

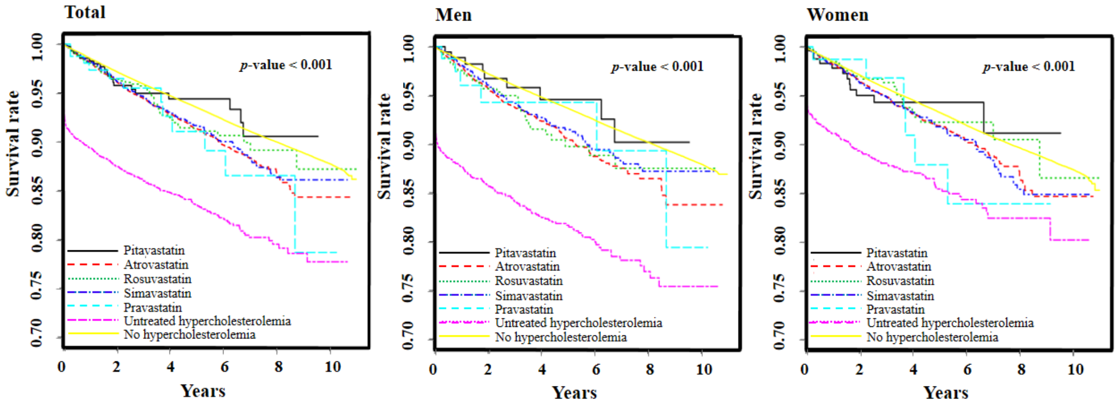

Figure S2. Kaplan-Meier estimates for development of cardio-cerebrovascular diseases and related deaths according to statin usage among individuals regardless of diabetes.

2C. Cardiovascular diseases, only

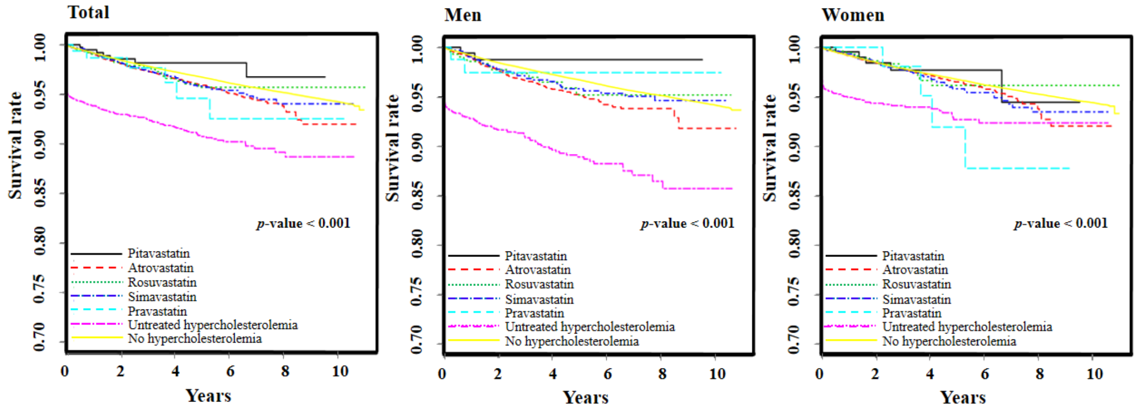

Figure S2. Kaplan-Meier estimates for development of cardio-cerebrovascular diseases and related deaths according to statin usage among individuals regardless of diabetes.

2D. Cerebrovascular diseases, only

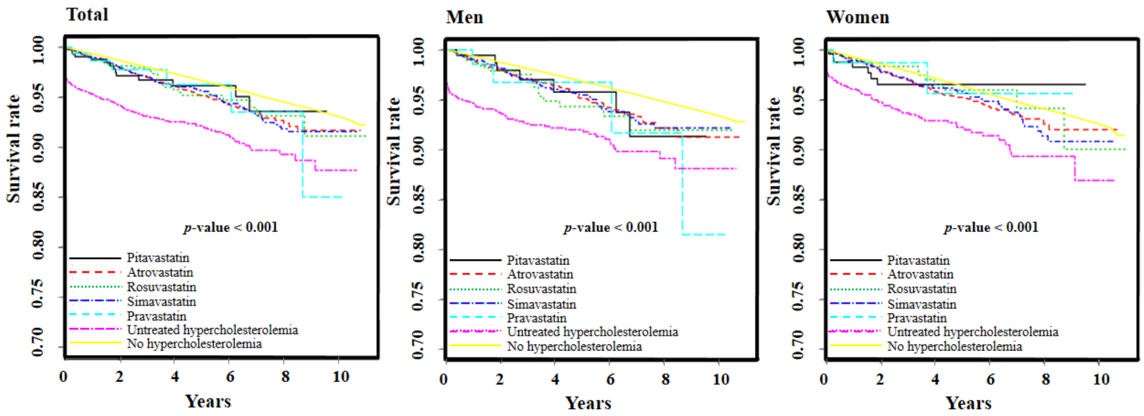

Figure 2. Kaplan-Meier estimates for development of cardio-cerebrovascular diseases and related deaths according to statin usage among individuals regardless of diabetes.

Table S3. Cox-proportional hazard regression model for composite outcomes (cardio-cerebrovascular diseases and related deaths) among individuals regardless of diabetes, compared with pitavastatin.

**Model**	**HRs (95% CIs)**	**Men**	**Women**
Model 1	Atorvastatin	0.977 (0.601–1.588)	1.128 (0.662–1.921)
	Rosuvastatin	1.013 (0.583–1.761)	0.990 (0.538–1.824)
	Simvastatin	0.886 (0.531–1.477)	1.257 (0.725–2.181)
	Pravastatin	1.049 (0.453–2.431)	1.293 (0.497–3.364)
	Untreated hypercholesterolemia	2.587 (1.588–4.215)	2.552 (1.485–4.385)
	No hypercholesterolemia	0.705 (0.438–1.135)	0.975 (0.577–1.648)
Model 2	Atorvastatin	0.957 (0.589–1.557)	1.129 (0.663–1.924)
	Rosuvastatin	1.001 (0.576–1.740)	0.992 (0.539–1.828)
	Simvastatin	0.860 (0.516–1.433)	1.255 (0.724–2.178)
	Pravastatin	1.006 (0.434–2.330)	1.299 (0.499–3.380)
	Untreated hypercholesterolemia	2.502 (1.536–4.077)	2.547 (1.482–4.377)
	No hypercholesterolemia	0.692 (0.430–1.114)	0.974 (0.577–1.646)
Model 3	Atorvastatin	0.960 (0.590–1.561)	1.113 (0.654–1.896)
	Rosuvastatin	1.011 (0.581–1.758)	0.997 (0.541–1.836)
	Simvastatin	0.872 (0.523–1.454)	1.248 (0.720–2.166)
	Pravastatin	1.029 (0.444–2.386)	1.278 (0.491–3.325)
	Untreated hypercholesterolemia	2.600 (1.596–4.237)	2.547 (1.482–4.377)
	No hypercholesterolemia	0.657 (0.408–1.059)	0.892 (0.527–1.508)

Model 1: adjusted for age; Model 2: adjusted for smoking status (ever and never smokers), drinking status (rare, sometimes, and often) and physical activity (rare, sometimes, and regular) in addition to variable of Model 1; Model 3: adjusted for body mass index, systolic blood pressure, total cholesterol, ALT, economic status (low, middle, and high), and diabetes (yes or no), in addition to variables of Model 2.
